# The Influence of the COVID-19 Outbreak on the Lifestyle of Older Patients With Dementia or Mild Cognitive Impairment Who Live Alone

**DOI:** 10.3389/fpsyt.2020.570580

**Published:** 2020-10-30

**Authors:** Mamoru Hashimoto, Maki Suzuki, Maki Hotta, Aki Nagase, Yuki Yamamoto, Natsuho Hirakawa, Yuma Nagata, Yuto Satake, Takashi Suehiro, Hideki Kanemoto, Kenji Yoshiyama, Etsuro Mori, Manabu Ikeda

**Affiliations:** ^1^Department of Psychiatry, Course of Integrated Medicine, Osaka University Graduate School of Medicine, Osaka, Japan; ^2^Department of Behavioral Neurology and Neuropsychiatry, Osaka University United Graduate School of Child Development, Osaka, Japan

**Keywords:** COVID-19, dementia, mild cognitive impairment, living alone, stay at home

## Abstract

**Background:** Under the COVID-19 outbreak, the Japanese government has strongly encouraged individuals to stay at home. The aim of the current study was to clarify the effects of the COVID-19 outbreak on the lifestyle of older adults with dementia or mild cognitive impairment (MCI) who live alone.

**Methods:** Seventy-four patients with dementia or MCI aged ≥65 years, who regularly visited the dementia clinic of the Department of Psychiatry, Osaka University Hospital, were recruited in this study. The patients were divided into two groups according to their living situation: living alone group (*n* = 12) and living together group (*n* = 62). Additionally, the spouses of patients aged ≥65 years were assigned to the healthy control group (*n* = 37). Subjects' lifestyle changes were evaluated between April 8 and 28, 2020.

**Results:** No subjects with acquaintances or relatives were infected with COVID-19 within the study period. The proportion of subjects who reduced going out in the living alone group, living together group and healthy control group was 18.2, 52.5, and 78.4%, respectively. The proportion of subjects who went out less frequently was significantly lower in both the living alone (*p* < 0.01) and living together (*p* < 0.05) groups than in the healthy control group.

**Conclusion:** Most patients with dementia or MCI who live alone did not limit their outings or activities during the COVID-19 outbreak. Regular monitoring for potential COVID-19 infection in people living alone with dementia is vital for their safety and well-being.

## Introduction

In Japan, an emergency declaration was issued, mainly in metropolitan areas, on April 7, 2020, because of the rapid increase in the number of patients affected by COVID-19. Under the declaration, the Japanese government urged the closure of non-essential businesses, schools and recreational facilities and strongly encouraged individuals to “stay at home,” except when doing essential activities.

Older adults and individuals with serious underlying medical conditions are thought to be at higher risk of severe illness from COVID-19 (1). With the rapid aging of the Japanese society and the increasing proportion of nuclear families in Japan, the number of older people living alone with dementia is increasing (2). Dementia affects various brain functions and is associated with impaired judgment and decision-making (3); thus, individuals with dementia may not take appropriate safety and preventive measures against COVID-19 because of inadequate understanding of the risks, which could result in problems regarding safety, particularly among those who live alone. A previous study reported that perception of fewer social resources and worse cognitive performance are risk factors for harm in people with dementia who are living alone (4). Other studies have suggested that people with dementia who live alone are at higher risk of adverse outcomes, such as malnutrition and weight loss, than those living with others (5, 6). These findings suggest that patients living alone with dementia may require special care during the COVID-19 outbreak.

In this study, we aimed to answer the following questions: Are people with dementia changing their lifestyle amidst the COVID-19 outbreak? Are they feeling stressed about their current situation? Do they have physical symptoms, such as sleep disorders or loss of appetite? Are these changes more pronounced in patients with dementia who live alone?

## Materials and Methods

### Subjects

This study was a prospective hospital-based cohort study. Subjects were recruited from those who regularly visited the dementia clinic of the Department of Psychiatry, Osaka University Hospital. All patients were examined comprehensively by psychiatrists (MHa, YS, TS, HK, KY, MI) and neurologists (EM) with sufficient experience in assessing patients with dementia. All patients underwent routine laboratory tests; standard neuropsychological and neurobehavioral examinations, including the Mini-Mental State Examination (MMSE) (7) and Clinical Dementia Rating (CDR) (8); and brain magnetic resonance imaging at the first visit. The diagnosis of each type of dementia and mild cognitive impairment (MCI) was established according to international consensus criteria. Specifically, the diagnoses of Alzheimer's disease (AD), dementia with Lewy bodies (DLB), frontotemporal dementia (FTD), and MCI were based on the NIA-AA criteria for probable AD (9), the revised consensus criteria for probable DLB in 2017 (10), the consensus diagnostic criteria for behavioral variant FTD (11) and the consensus clinical diagnostic criteria in an international workshop for semantic dementia (12), and the criteria for MCI of Petersen's criteria (13), respectively. Consecutive patients with dementia or MCI who had a telephone visit or an outpatient visit to our dementia clinic between April 8 and 28, 2020 were included in this study. We set a short-term survey period of 3 weeks from the day after the emergency declaration to identify the short-term influence of the COVID-19 outbreak on the lifestyle of patients with dementia. The exclusion criteria were as follows: (1) patients aged <65 years, (2) patients with severe dementia (CDR 3), (3) patients who did not undergo MMSE within the last year, (4) patients in a nursing home, (5) patients without a reliable informant, and (6) patients who were unable to provide informed consent.

Patients with dementia or MCI were divided into two groups according to living situation: living alone group and living together group. Those who live with their families were assigned to the living together group. Additionally, the spouses of the patients aged ≥65 years were used as the healthy control group. If there was a cohabitant other than the couple, the spouse was excluded from the healthy control group.

All procedures followed the Clinical Study Guidelines of the Ethics Committee of Osaka University, Osaka, Japan, and were approved by the internal review board. After a complete description of all procedures in the study, informed consent was obtained from the patients and/or their caregivers in compliance with the research standards for human research and in accordance with the Declaration of Helsinki.

### Procedures

We evaluated the physical and mental conditions and lifestyle changes of the subjects during the COVID-19 outbreak using an original questionnaire (Table 1). In this study, we created a new questionnaire that could be easily and quickly conducted, even by telephone, although its validity and reliability have not been verified. Caregivers and/or patients were asked questions by the medical staff, including neuropsychologists (MS, YY, and NH), occupational therapists (MHo and YN), and a geriatric nurse (AN), at the time of the consultation, either by in-person interview or by telephone during the survey period. The current health status compared with that in December was assessed. Moreover, the respondents were instructed to answer “yes,” “no,” or “don't know” to each question. The “don't know” responses were not considered in the analyses. To compare the rates of subjects who answered “yes” to each question among the living alone, living together, and healthy control groups, we used the χ^2^-test with Fisher's exact probability test and performed residual analysis using the Bonferroni *z*-test for each comparison when the overall group difference was significant. The statistical threshold was set at *p* < 0.05. All analyses were performed using SPSS version 25.0 (SPSS Inc., Chicago, IL, USA).

**Table 1:** Lifestyle changes questionnaire.

**Please tell us about the patient's current state compared with that in December**
1.Is there any change in how the patient spend his or her days?
2.Is the patient going out less frequently?
3.Is the patient spending more time at home?
4.Is the patient engaging in less activity or exercise?
5.Is the patient taking more naps?
6.Did the COVID-19 outbreak increase the patient's mental stress?
7. Has the patient lost his or her appetite?
8.Is the patient's appetite increasing?
9.Does the patient have difficulty sleeping?
10.Has the patient had constipation?/Is the constipation worse?

*When asking the healthy control group, we replaced “the patient” with “you” in each question*.

## Results

Twelve patients who live alone, 62 patients who live together with their families, and 37 caregivers participated in this study. Table 2 shows the demographics of the subjects. We used the MMSE and CDR scores that were obtained within the year. A significant difference in sex and age among the three groups was found. The proportion of men was significantly lower in the living alone group than in the living together group (p < 0.05) and the healthy control groups (*p* < 0.01). Patients in the living alone group were significantly older than those in the living together group (*p* < 0.01) and the healthy control group (*p* < 0.01). No significant differences in the MMSE scores (*p* = 0.955) and the proportion of patients using care services (*p* = 0.352) between the living alone and living together groups were observed.

**Table 2:** Subjects' demographics.

	**Living alone group (*n* = 12)**	**Living together group (*n* = 62)**	**Healthy control group (*n* = 37)**	***p*-value**	***Post-hoc* test**
Male/Female	1/11	26/36	22/15	0.007^a^	Living together, Control > Living alone
Age (years)	80.9 ± 7.9	75.4 ± 6.3	74.8 ± 5.7	0.012^b^	Living alone > Living together, Control
MMSE score	20.3 ± 4.8	20.4 ± 7.0	n.a.	0.955^c^	n.a.
CDR (0.5/1/2)	7/4/1	29/27/6	n.a.	0.622^d^	n.a.
Use of nursing care service	7 (58%)	26 (42%)	n.a.	0.352^a^	n.a.
**Disease**					
AD	6 (50%)	25 (40.3%)	n.a.	0.543^a^	n.a.
DLB	0	7 (11.3%)	n.a.	0.590^a^	n.a.
FTD	3 (25%)	8 (12.9%)	n.a.	0.371^a^	n.a.
MCI	3 (25%)	14 (22.6%)	n.a.	1.00^a^	n.a.
Others	0	8 (12.9%)	n.a.	0.339^a^	n.a.

*Values are n or mean ± SD*.

*MMSE, Mini-mental State Examination; CDR, Clinical Dementia Rating; AD, Alzheimer's Disease; DLB, Dementia with Lewy bodies; FTD, frontotemporal dementia; MCI, mild cognitive impairment; n.a., not applicable*.

a *Fisher's exact probability test*,

b *One-way analysis of variance*,

c *t-test*,

d *Mann-Whitney U-test*.

Table 3 shows the positive response rate for each question in the three groups. Significant group differences were observed for the positive response rates for “change in how to spend the day” (*p* < 0.01), “decrease in going out” (*p* < 0.001), “increase in staying at home” (*p* < 0.001), and “increase in mental stress” (*p* < 0.001). *Z*-tests showed that the positive response rate for “change in how to spend the day” was significantly higher in the healthy control group than in the living alone group (*p* < 0.01). The positive response rate for “decrease in going out” was significantly higher in the healthy control group than in the living alone (*p* < 0.01) and living together (*p* < 0.05) groups. The positive response rate for “increase in staying at home” was significantly higher in the living together (*p* < 0.01) and the healthy control (*p* < 0.001) groups than in the living alone group. The positive response rate for “increase in mental stress” was significantly higher in the healthy control group than in the living alone (*p* < 0.01) and living together (*p* < 0.001) groups. The subjects and their family or relatives were not infected with COVID-19 as confirmed by PCR test within the study period.

**Table 3:** Positive response rate for each question in the three groups.

	**Living alone group (*n* = 12) (%)**	**Living together group (*n* = 62) (%)**	**Healthy control group (*n* = 37) (%)**	***p*-value**	***Z*-test with Bonferroni correction**
1. Change in how to spend the day	16.7	50.8	70.3	0.004	Living alone < Control
2. Decrease in going out	18.2	52.5	78.4	0.001	Living alone, Living together < Control
3. Increase in staying at home	9.1	56.5	77.8	<0.001	Living alone < Living together, Control
4. Decrease in activity or exercise	16.7	54.9	47.2	0.053	n.a.
5. Increase in nap	25	22.6	11.1	0.326	n.a.
6. Increase in mental stress	9.1	18.6	58.3	<0.001	Living alone, Living together < Control
7. Loss of appetite	0	1.7	11.1	0.078	n.a.
8. Increase in appetite	0	15	2.8	0.068	n.a.
9. Sleeping disorder	33.3	17.9	40.7	0.061	n.a.
10. Constipation	9.1	13.0	10.8	0.972	n.a.

*Analysis by χ^2^-test with Fisher's exact probability test and Bonferroni z-test*.

*n.a., not applicable*.

## Discussion

The major finding of this study is that most patients with dementia or MCI who live alone did not limit their outings or activities during the COVID-19 outbreak, whereas more than half of the patients who live together with their families reduced their frequency of going out. This finding may be attributed to the need of the patients living alone to go out for shopping; thus, they had to go out more often than those living together with their families. However, nearly 80% of healthy older adults who were caregivers of patients with dementia in this study reduced their frequency of going out, despite the need to go out for essential items. Hence, the reason why patients with dementia who live alone did not restrict their outings may be mainly attributed to poor recognition of the risk of COVID-19 infection, which could be associated with cognitive decline, rather than the need to go out. Additionally, patients who live alone had no caregivers nearby to encourage them to stay at home, which may also have an effect on their behavior. A previous study reported that worse cognitive performance is a risk factor for harm among people with dementia who are living alone (4). Therefore, regular monitoring for potential COVID-19 infection among people with cognitive impairment who are living alone appears to be vital for their safety and well-being.

During this COVID-19 pandemic, “stay at home” has been used as a public health slogan by the Japanese government. Consequently, numerous residents are experiencing social isolation, which could result in physical and psychological health issues, particularly among patients with dementia and their caregivers. More than 70% of the caregivers reported a reduced frequency of going out, and nearly 60% felt psychological stress. Conversely, patients with dementia or MCI reported significantly less psychological stress than caregivers, regardless of living conditions. Additionally, the results revealed that few patients with dementia had mental and physical changes such as insomnia or changes in appetite. Patients with dementia, particularly those living alone, exhibited little change in their lifestyle, which may have influenced the current results. Another possibility is that significant effects, such as mental stress, may have not yet emerged in patients with dementia during the survey, which was conducted shortly after the emergency declaration was made. Moreover, the mental stress of the patients may have been underestimated because the information in this study was mainly obtained from caregivers. However, our results suggest the urgent need for support for caregivers of people with dementia, as recommended by international dementia experts and Alzheimer's Disease International (14).

The demographic characteristics of the subjects who live alone were different from those of individuals who live with their families. Differences in the background characteristics, such as dementia type, sex, and age, between the groups may have influenced the results. The living alone group included three patients with FTD (25%), while the proportion of patients with FTD in the living together group was 12.9%. Patients with FTD tend to show distinctive unusual behaviors, such as disinhibition, loss of social awareness, and stereotyped behavior (11), which could make it difficult for them to adapt the drastic changes in lifestyle caused by the COVID-19 outbreak (15). The higher proportion of patients with FTD in the living alone group may have resulted in the higher frequency of going out. Regarding sex, the number of males was significantly lower in the living alone group than that in the living together group, which is consistent with previous reports (5, 16). Men, especially those who belong to the older generations in Japan, are less likely to be involved in housekeeping activities, such as shopping and cooking (17). Thus, the higher proportion of men in the living together group, who did not usually go shopping, may have contributed to the lower frequency of going out. Moreover, patients in the living alone group were significantly older than those in the living together and healthy control groups. Although the role of age in the ability of people with dementia to adapt to environmental changes remains unclear, age difference among the three groups in this study possibly influenced the results.

Several methodological issues limit the interpretation of our results. First, the number of patients living alone was small (*n* = 12) because we set a short-term survey period of 3 weeks. Thus, the severity of cognitive dysfunction, which could affect the lifestyle of patients with dementia, was not considered in our study. Second, we used an original questionnaire in this study, which has not been validated for reliability or validity. Third, we did not investigate the support of family and friends, which could affect the lifestyle of patients living alone. Nonetheless, no significant difference in the frequency of use of nursing care services between the living alone group and the living together group was found. Further investigations are needed to address this issue.

In conclusion, most of the patients with dementia or MCI who live alone in this study did not limit their outings or activities during the COVID-19 outbreak. Regular monitoring for potential COVID-19 infection among these patients is vital for their safety and well-being.

## Data Availability Statement

The datasets presented in this article are not readily available because Research data are not shared. Requests to access the datasets should be directed to Mamoru Hashimoto, mhashimoto@psy.med.osaka-u.ac.jp.

## Ethics Statement

The studies involving human participants were reviewed and approved by the Ethics Committee of Osaka University. The patients/participants provided their written informed consent to participate in this study.

## Author Contributions

MHa contributed to the study concept, review of the literature, and writing of the manuscript. MS was involved in supervising the analysis and writing of the manuscript. MHo and AN contributed to the study design, data acquisition, data collection, and review of the manuscript. YY, NH, YN, YS, TS, HK, and KY were involved in the collection of data and review of the manuscript. EM was responsible for the critical review of the manuscript and the study design. MI contributed to the study concept, review of data, and review of the manuscript. All authors contributed to the article and approved the submitted version.

## Conflict of Interest

The authors declare that the research was conducted in the absence of any commercial or financial relationships that could be construed as a potential conflict of interest.
